# Effects of Cerebellar tACS With Gamma Band on Basketball Shooting Skills: A Single‐Blind, Randomized Controlled Trial in College Students With Basketball Experience

**DOI:** 10.1002/brb3.70943

**Published:** 2025-10-15

**Authors:** Shota Miyaguchi, Yasuto Inukai, Miyu Muroi, Shunpei Yamamoto, Hideaki Onishi

**Affiliations:** ^1^ Institute for Human Movement and Medical Sciences Niigata University of Health and Welfare Niigata Japan; ^2^ Department of Physical Therapy Niigata University of Health and Welfare Niigata Japan; ^3^ Graduate School, Niigata University of Health and Welfare Niigata Japan

**Keywords:** basketball shooting skill, cerebellum, transcranial alternating current stimulation

## Abstract

**Introduction:**

Transcranial electrical stimulation (tES) is a widely used noninvasive brain stimulation method to improve motor performance. Transcranial alternating current stimulation (tACS), which modulates oscillatory brain activity, has been extensively investigated as a tES method to enhance motor skills. However, few studies have investigated the effects of tACS on sports performance. The purpose of this study was to determine the effects of cerebellar tACS on basketball shooting skills in college students with basketball experience. This study was conducted as a single‐blind, randomized controlled trial.

**Methods:**

A total of 36 healthy young women (average age: 20.2 years) who were with former basketball players were included in the study. tACS (1.0 mA, 70 Hz) was administered for 15 min with 25 cm^2^ electrodes placed on the scalp over the bilateral cerebellar hemispheres. The stimulus frequency was selected based on prior findings showing effectiveness in gamma‐band modulation. Shooting skills were assessed with a two‐handed free throw shooting task. Shooting skills were scored on a 6‐point scale per shot, and 30 shots (10 shots × 3 sets) were taken before and after the tACS intervention. This task has been validated as a reliable measure of shooting accuracy in previous studies.

**Results:**

Shooting scores significantly increased after stimulation in the tACS group (Median [IQR]: 34.8 [27.3–37.9]–37.0 [30–40.1], *p* = 0.028, *r_rb* = −0.661); however, no significant differences in shooting scores were detected before and after stimulation in the sham group (33 [29.9–36.9]–32 [25.4–36.9], *p* = 0.310, *r_rb* = 0.404). There was no significant change in shooting success rates before and after the intervention in either group (tACS group: 62.5 [38.8–75]–65 [43.8–76.3], *p* = 0.751, *r_rb* = −0.258, sham group: 57.5 [45–76.3]–55 [26.3–67.5], *p* = 0.810, *r_rb* = 0.235).

**Conclusions:**

Our results indicate that 70 Hz tACS over the cerebellum may improve basketball shooting skills. These results provide valuable insights into the practical application of tACS in sports.

l The purpose of this study was to determine the effects of cerebellar tACS on basketball shooting skills.
l A total of 36 healthy young women with basketball experience received cerebellar tACS (1.0 mA, 70 Hz) for 15 min.
l Shooting scores significantly increased after cerebellar tACS intervention.

## Graphical abstract

- The purpose of this study was to determine the effects of cerebellar tACS on basketball shooting skills.
- Thirty‐six healthy young women with basketball experience received cerebellar tACS (1.0 mA, 70 Hz) for 15 minutes.
- Shooting scores significantly increased after cerebellar tACS intervention.

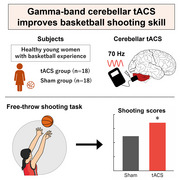

## 1 Introduction

Transcranial electrical stimulation (tES) is a widely used noninvasive brain stimulation method to improve motor performance (Machado et al. 2019; Wang et al. 2021). The most common tES technique is transcranial direct current stimulation (tDCS), and its effects on sports performance have been widely investigated (Maudrich et al. 2022). For example, some studies have reported that tES enhances sports performance, such as improvements in shooting and dribbling in basketball (Veldema et al. 2022; Moscaleski et al. 2022), passing accuracy after soccer matches (Shiravand et al. 2024), spike speed and stability in volleyball (S. B. Park et al. 2023), selective attention and reaction time in boxing (Kamali et al. 2021), endurance when using an ergometer (Pollastri et al. 2021), and lower‐limb power during jump tasks (Grosprêtre et al. 2021). However, an umbrella review found no strong evidence that acute tDCS significantly enhances sports performance (Holgado et al. 2024). Therefore, evidence regarding the effects of tDCS on sports performance remains limited, and no consistently effective stimulation method has been established. In recent years, multiple tES methods have been developed. Evaluating the effects of tES using various stimulation methods may help validate its potential to improve sports performance.

Transcranial alternating current stimulation (tACS), another tES method, modulates oscillatory brain activity to enhance motor skills (Takeuchi and Izumi 2021; Hu et al. 2022). During tACS, a weak alternating current is applied through electrodes placed on the scalp to modulate oscillatory neural activities in targeted areas (Herrmann et al. 2013; Antal and Herrmann 2016). Notably, tACS modulates brain oscillations through an entrainment mechanism, where the phase of the oscillatory activity shifts in response to the frequency of the applied alternating current (Reato et al. 2013). The entrainment effects of oscillatory neural activities persist for 70 min after the stimulation is terminated (Helfrich et al. 2014; Kasten et al. 2016). Given that brain oscillation frequencies, such as alpha (8–12 Hz), beta (13–30 Hz), and gamma (30–100 Hz), contribute to motor modulation and learning, these frequencies are commonly used in tACS studies (McNally et al. 2024; Wiesman et al. 2021). Specifically, gamma‐band activity in the primary motor cortex (M1) increases during motor execution, and stimulation at gamma frequencies is often used to enhance motor function (McNally et al. 2024). Previous studies have demonstrated that gamma‐band tACS improves motor skills (Rostami et al. 2023), and we have also shown that 70‐Hz gamma‐band tACS augments motor function (Miyaguchi, Inukai, Mitsumoto, et al. 2022; Miyaguchi et al. 2020). However, most of these studies focused on fine motor control tasks in laboratory settings; very few studies focused on the effects of tACS on complex motor tasks involving the whole body, such as sports performance. Giustiniani et al. (2021) showed that applying 50‐Hz tACS over bilateral M1 did not affect jump performance or upper body power. In addition, Wilkins et al. (2024) showed that applying 70‐Hz tACS to M1 did not improve performance in an overhand throw task. As few studies have examined the effects of tACS over M1 on sports performance, stimulation at other frequencies, such as alpha or beta, may also be beneficial. However, considering the limited evidence currently available, applying tACS to M1 has not been shown to improve sports performance. Because sports performance requires precise, full‐body control, it involves not only M1 but also other cortical regions. Therefore, targeting cortical areas other than M1 for stimulation may be a valid and effective approach.

In addition to M1, tACS over the cerebellar region has been investigated (Miyaguchi et al. 2020). Gamma‐band tACS over the cerebellum modulated Purkinje cell activity and cerebellar nuclei neurons in animal experiments (Asan et al. 2020; Mourra et al. 2025). Gamma‐band tACS targeting the cerebellar region can enhance finger motor function (Miyaguchi, Inukai, Mitsumoto, et al. 2022; Naro et al. 2017). In addition, 70‐Hz tACS over the cerebellum improves bimanual motor skills (Miyaguchi, Inukai, Mitsumoto, et al. 2022), and stimulating the cerebellum was more effective than stimulating the M1. The cerebellar region is involved in various functions necessary for sports skills, including motor learning, precise motor adjustments using feedback, and body balance (Ito 2000; King et al. 2019). Therefore, tACS interventions targeting the cerebellum may enhance sports performance. However, the effects of tACS over the cerebellum on sports performance have not been investigated.

The objective of this study was to determine the effects of tACS over the cerebellum on basketball shooting skills in young amateur women. Basketball players have larger gray matter volumes in cerebellar lobes VI and VII, suggesting that these regions support hand dexterity and bimanual coordination, which are necessary for shooting and dribbling (I. S. Park et al. 2009, 2018). Previous research showed that gamma‐band tACS over the cerebellum improves fine motor skills and bimanual motor skills (Miyaguchi, Inukai, Mitsumoto, et al. 2022; Naro et al. 2017). Therefore, the effects of cerebellar tACS on shooting skills with both hands were examined. We hypothesized that the cerebellum enhances precise motor adjustments using visual feedback and finger dexterity to improve shooting skills with both hands. The results of this study will provide fundamental insights into the practical application of tACS in sports.

## 2 Materials and Methods

### 2.1 Subjects

A total of 36 healthy young women (mean age: 20.2 ± 0.9 years) with basketball‐playing experience were included in this study. Participants were recruited through a student social networking platform at the researchers’ university, and all subjects voluntarily participated in the study. The subjects’ basketball training level was amateur; they had at least 3 years of club experience, and they were not currently training habitually. As we previously showed that cerebellar tACS improves bimanual hand dexterity, we also chose a bimanual motor task for the present study (Miyaguchi, Inukai, Mitsumoto, et al. 2022). This study focused on female subjects because female basketball players shoot with two hands during games. Subjects were randomly assigned to the tACS or sham groups (*n* = 18 per group) using the RAND function in Excel. The number of subjects was determined using G*Power 3.1.9.7 for the effect size (*d*) of 0.25 and a statistical power (1 − β) of 0.80. All subjects reported no history of neurological or psychiatric diseases or orthopedic diseases under treatment, all subjects were medication‐free during the study, and all subjects had normal or corrected‐to‐normal vision. The subjects had no contraindications to tES (i.e., no metal implants in the head, pacemakers, seizures, or scalp or head injuries). The study was approved by the Ethics Committee of the Niigata University of Health and Welfare (19441‐241213) and was conducted in accordance with the principles of the Declaration of Helsinki. Written informed consent was obtained from each subject. Additionally, subjects received monetary compensation after the experiment.

### 2.2 Basketball Shooting Skills

**FIGURE 1:**
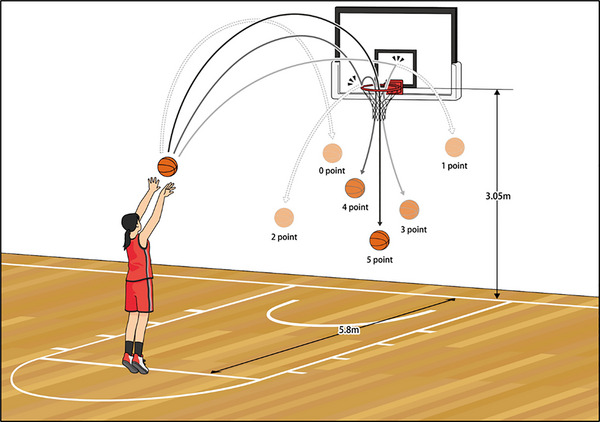
Free throw shooting task. The subject shot from the free throw line with both hands. Free throw shots were graded as 5 points if the ball entered the basket without hitting the ring, 4 points if the ball hit the ring and entered the basket, 3 points if the ball hit the board and entered the basket, 2 points if it hit the ring and missed, 1 point if it hit the board and missed, and 0 points if it hit nowhere and missed. To standardize the free throw shooting task, participants followed the following five instructions: (1) position yourself in front of the hoop, (2) grip the ball with both hands, (3) focus on the hoop for at least 3 s before shooting, (4) avoid jumping, and (5) aim to make the basket without the ball touching the rim or backboard.

The free throw shooting task was used to assess basketball shooting skills in this study. Figure 1 shows the free throw task. Subjects shot internationally sanctioned basketballs (BGL6, Molten) from the free throw line. The free throw line and the height of the ring (3.05 m) complied with the International Basketball Federation. To standardize the shooting behavior of the free throw task, the following five instructions were given to each subject: (1) stand in front of the ring, (2) hold the ball with both hands, (3) look at the ring for more than 3 s before shooting, (4) do not jump, and (5) try to make the basket without hitting the ring and board. The participants were also asked to watch a video of an experienced player executing a free throw just before the shooting skills evaluation to fully understand the shooting motion. The examiner monitored the participants to ensure that the instructions were followed and the shooting motion was performed correctly. free throw attempts were scored as follows: 5 points if the ball entered the basket without hitting the ring, 4 points if the ball hit the ring and entered, 3 points if the ball hit the board and entered, 2 points if the ball hit the ring and missed, 1 point if the ball hit the board and missed, and 0 points if the ball did not hit anywhere and missed (Figure 1). Our previous studies established that the scoring system accurately assessed motor learning of basketball shooting skills (Miyaguchi, Inukai, Mitsumoto, et al. 2022; Miyaguchi, Inukai, Hashimoto, et al. 2022).

### 2.3 Transcranial ACS

**FIGURE 2:**
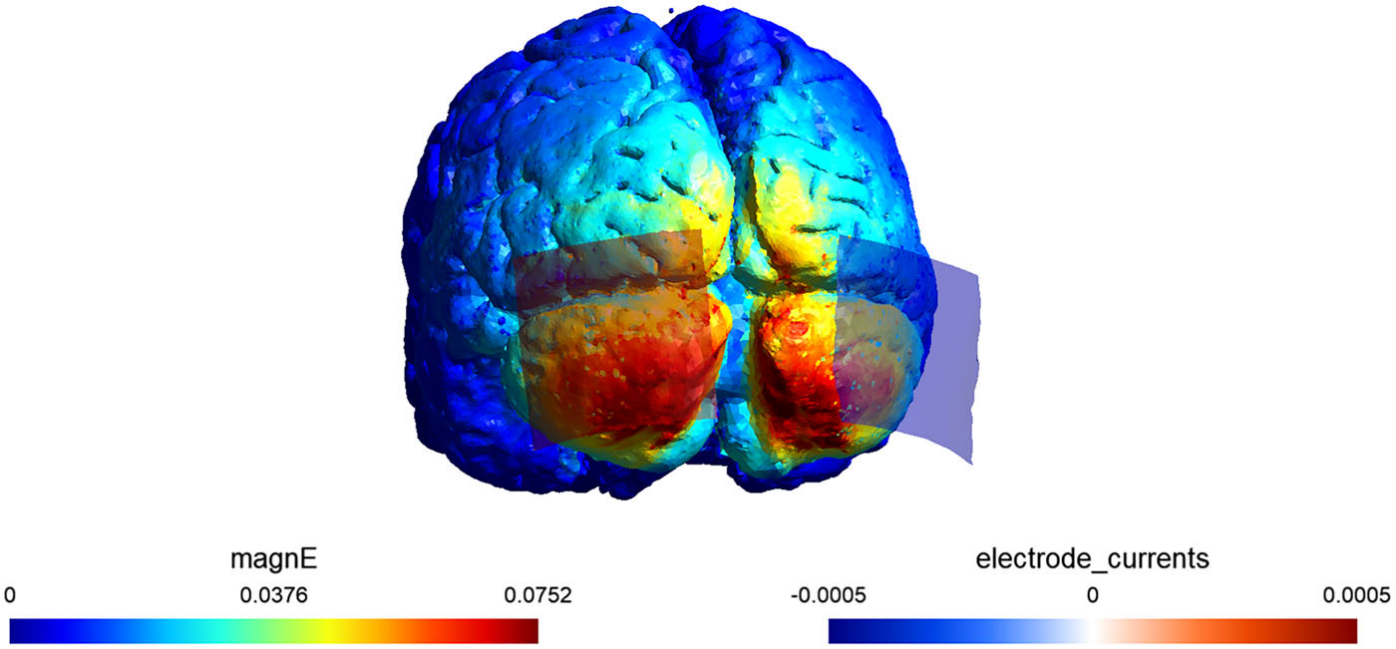
Electrode montage and SimNIBS version 4.0.1 electric field simulations.

The tACS was administered with a DC‐stimulator (Eldith, neuroConn GmbH, Ilmenau, Germany) via two saline‐soaked surface sponge electrodes. The 25 cm^2^ (5 cm × 5 cm) electrodes were placed on the scalp over the cerebellum. The center of the cerebellum electrodes was placed on the subject's scalp 2.0 cm below I1 and I2 in the international 10–10 method. The following stimulus parameters were used: intensity 1.0 mA (peak‐to‐peak current), frequency 70 Hz in the gamma band, duration 15 min, and fade‐in/out cycles of 5 s. These tACS stimulus parameters modulated motor performance in previous studies (Miyaguchi, Inukai, Mitsumoto, et al. 2022). Electric field simulations were conducted using SimNIBS version 4.0.1, and the generation of a localized electric field in the cerebellar region was confirmed (Figure 2). In the sham group, cerebellar stimulation was performed for 10 s of fade‐in/out at 70 Hz. The application of tACS complied with safety guidelines (Antal et al. 2017). To examine the side effects of tACS, including phosphenes, itching, headache, burning sensation, and dizziness, the subjects rated the intensity of their side effects on a 7‐point scale (0 = *no sensations* to 6 *= very strong sensations*) (Raco et al. 2014) (Supporting Information). Side‐effect intensity was assessed at three time points: immediately after tACS stimulation began (0 min), during tACS intervention (7.5 min from the start), and immediately after tACS intervention (15 min from the start). The tACS intervention was conducted using a single‐blind protocol for participants. After the experiment, to assess whether they could distinguish real from sham stimulation, participants were asked which condition they thought they had received. They were then informed of the correct condition. The discrimination rate (number of correct responses/number of participants in the group × 100) was then calculated for each group.

### 2.4 Experimental Procedure

**FIGURE 3:**
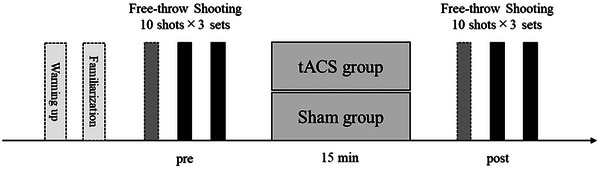
Experimental procedure.

Figure 3 shows the experimental procedure. First, the subjects jogged for 3 min to warm up. Then, the subjects shot one free throw shoting as a familiarization exercise to confirm their understanding of the task. The subjects performed the free throw shoot task (10 shots × 3 sets) before the tACS intervention. The first set was used as a warm‐up, and the latter two sets were evaluated as test trials. After the 15‐min tACS intervention, the participants performed the free throw shooting task again.

### 2.5 Statistical Analyses

The average prestimulus and poststimulus shoot scores and the average prestimulus and poststimulus shooting success rate (number of successful shots/total shots × 100) in each group were calculated. As data were not normally distributed according to the Shapiro–Wilk test, prestimulus and poststimulus scores were compared using the Wilcoxon signed‐rank test (Bonferroni correction). Subject information (age, height, weight, handedness, and competitive history) and the side effects scale were also not normally distributed and were compared between groups using the Mann–Whitney *U* test. Differences were considered statistically significant when *p* < 0.05. Cohen's *d* was used for the effect size of the parametric test, and Rank‐Biserial Correlation (*r_rb*) was used for the effect size of the nonparametric test. IBM SPSS Statistics version 24 software (IBM Corp., Armonk, NY, USA) was used for all statistical analyses.

## 3 Results

All 36 subjects completed the assessments without interruption. Table 1 shows the subject's age, height, weight, Edinburgh handedness score, and competition history. The Mann–Whitney *U* test did not show any significant differences in any participant information between the two groups (*p* > 0.5). The mean itching values are shown in Table 2. The Mann–Whitney *U* test did not reveal any significant difference in itching values between the two groups. No subjects complained of side effects other than itching. The percentages of participants reporting each side effect were as follows: phosphenes 0 %, headache 0 %, burning sensation 0 %, and dizziness 0 %. Furthermore, the stimulus discrimination rates were 44.4% for the tACS group and 50.0% for the sham group. In both groups, the stimulus discrimination rates were at approximately chance levels.

**TABLE 1:** Participant demographic information.

	tACS group	Sham group	Mann–Whitney *U* test
Age (years)	20.3 ± 1.0	20.1 ± 0.8	*p* = 0.606
Height (cm)	159.2 ± 6.5	159.0 ± 6.1	*p* = 0.501
Weight (kg)	52.8 ± 7.3	54.8 ± 8.4	*p* = 0.696
Handedness (%)	71.8 ± 47.8	73.8 ± 55.3	*p* = 0.719
Competitive history (years)	8.3 ± 3.1	8.8 ± 3.0	*p* = 0.628

*Note*: Mean ± standard deviations.

**TABLE 2:** Itching scores.

	tACS group	Sham group	Mann–Whitney *U* test
0 min	0.72 ± 1.07	0.50 ± 0.86	*p* = 0.584
7.5 min	0.11 ± 0.32	0.17 ± 0.71	*p* = 0.815
15 min	0.06 ± 0.24	0.17 ± 0.71	*p* = 0.988

*Note*: 0 = *no sensation* to 6 = *very strong sensation*.

**FIGURE 4:**
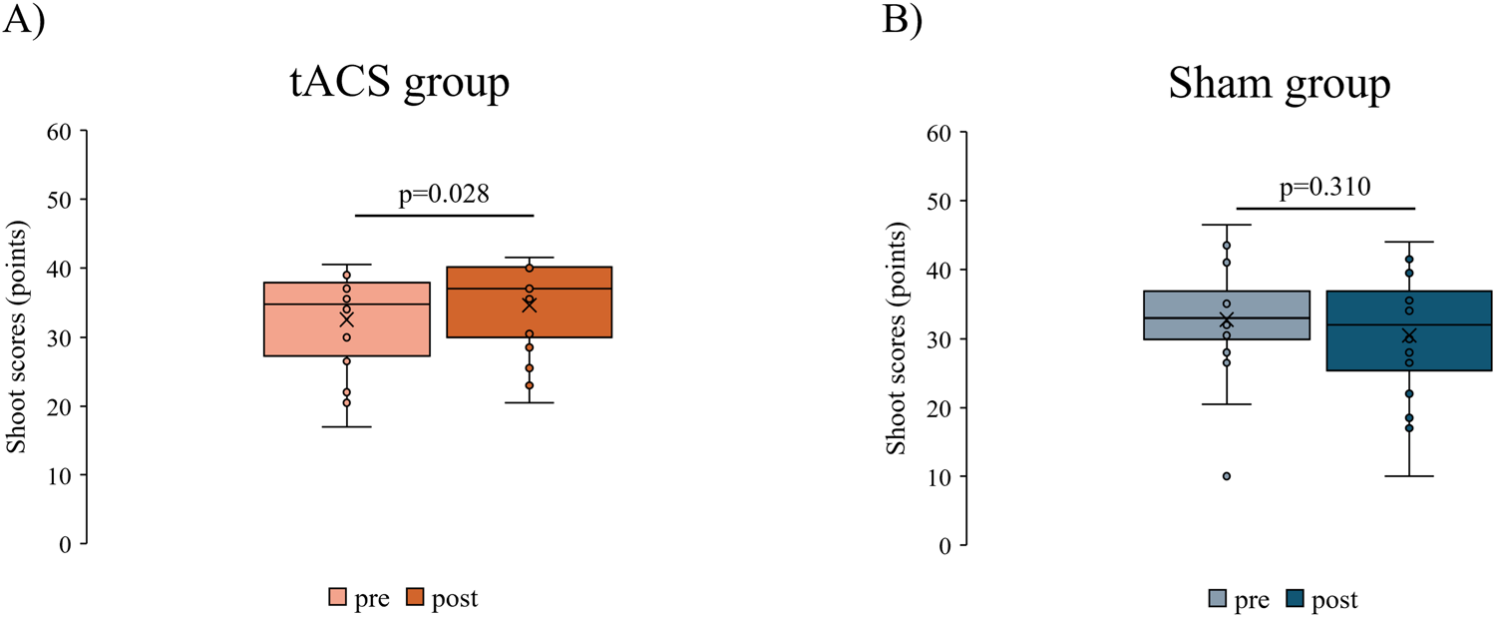
Shoot scores before and after tACS intervention in each group. (A) tACS group. (B) Sham group. The *p* value is shown using the Wilcoxon signed‐rank test (Bonferroni correction).

**FIGURE 5:**
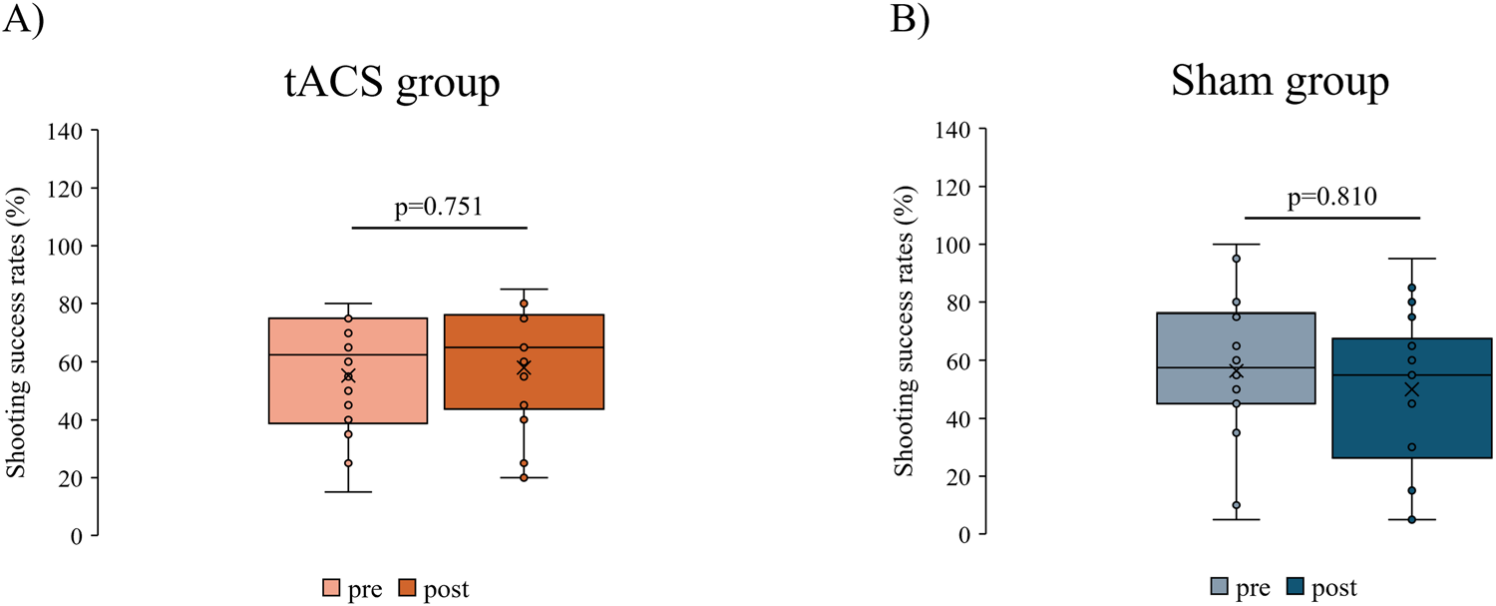
Shooting success rates before and after tACS intervention in each group. (A) tACS group. (B) Sham group. The *p* value is shown using the Wilcoxon signed‐rank test (Bonferroni correction).

Figure 4 shows the shoot scores for each group. The Mann–Whitney *U* test showed no significant difference in the shoot score at pre in each group (*p* = 0.938, Cohen's *d* = −0.028). The Wilcoxon signed‐rank test results showed a significant increase in the poststimulus shoot scores compared with the prestimulus score in the tACS group (Median [IQR]: 34.8 [27.3–37.9]–37.0 [30–40.1], *p* = 0.028, *r_rb* = −0.661) (Figure 4A). No significant changes in the shoot scores before and after stimulation were detected in the sham group (33 [29.9–36.9]–32 [25.4–36.9], *p* = 0.310, *r_rb* = 0.404) (Figure 4B). The percent change in the shoot score before and after stimulation in each group was 1.9% ± 4.3% in the tACS group and −2.3 ± 6.3% in the sham group. Figure 5 shows the shooting success rates for each group. The Mann–Whitney *U* test showed no significant difference in the shooting success rates at pre in each group (*p* = 0.963, Cohen's *d* = −0.048). The Wilcoxon signed‐rank test showed no change in the shooting success rate before and after stimulation in either group (tACS group: 62.5 [38.8–75]–65 [43.8–76.3], *p* = 0.751, *r_rb* = −0.258; sham group: 57.5 [45–76.3]–55 [26.3–67.5], *p* = 0.810, *r_rb* = 0.235). The percent change in the shooting success rate before and after stimulation in each group was 2.8% ± 15.3% in the tACS group and −6.4% ± 19.4% in the sham group.

## 4 Discussion

This study evaluated shooting scores and shooting success rates before and after 15‐min 70‐Hz tACS intervention over the cerebellum to investigate its effects on basketball shooting skills in healthy female adults. The results showed that tACS induced a significant increase in free shooting scores, but no difference before and after the intervention was observed in the sham group. We demonstrated that applying 70‐Hz tACS to the cerebellum for 15 min improves basketball shooting skill.

In this study, the scores for free throw shooting increased after cerebellar gamma‐band tACS. A previous study using transcranial magnetic stimulation demonstrated that gamma‐band tACS over the cerebellum improves finger dexterity by reducing cerebellar inhibition, specifically the inhibitory pathway from the cerebellum to the M1 (cerebellar inhibition) (Naro et al. 2017). Accordingly, the improvement in shooting scores observed after tACS in the present study may be due to changes in the activity of inhibitory interneurons, such as basket cells and stellate cells, Purkinje cells, and cerebellar nuclear cells in the cerebellum. However, as no neurophysiological measurements were taken in the current study, the discussion of underlying mechanisms remains speculative and based solely on interpretations drawn from previous research. Inhibitory interneurons in the cerebellar molecular layer are active at 30–80 Hz (Middleton et al. 2008) and connect inhibitory cells to Purkinje cells. Purkinje cells are the only output cells in the cerebellum; they inhibit excitability to M1 via the dentate nucleus and thalamus (Cheron et al. 2016). Disinhibition of the inhibitory circuit from the cerebellum to M1 caused by suppression of Purkinje cell activity may contribute to the improved motor skills induced by cerebellar tACS (Spampinato, Block, et al. 2017; Spampinato and Celnik 2017; Opie et al. 2022). In animal experiments, tACS (E‐field: 0.05–0.1 V/m) over the cerebellum affected the activity of Purkinje cells and cerebellar nuclear neurons in a frequency‐dependent manner in the frequency range of 0.5–80 Hz (Mourra et al. 2025). Transcranial ACS over the cerebellar cortex does not directly affect the cerebellar nuclear neurons but influences the neurons indirectly by modulating the activity of the upstream Purkinje cells. Therefore, adjusting Purkinje cell and cerebellar nuclear cell activity by cerebellar tACS enhances motor function. However, the neural mechanisms of cerebellar tACS in humans are unclear. The effects may vary depending on electrode placement and electric field strength; thus, further investigation is necessary. Previous behavioral experiments showed that 70‐Hz tACS over the cerebellum improves bimanual motor skills (Miyaguchi, Inukai, Mitsumoto, et al. 2022), and 50‐Hz tACS over the cerebellum enhances finger dexterity (Naro et al. 2017; Herzog et al. 2023). The results of this study agree with these prior findings. To the best of our knowledge, the effects of cerebellar tACS on sports skills have not been investigated. Thus, this is the first report demonstrating that cerebellar tACS enhances sports skills.

However, it is essential to interpret with caution the finding that cerebellar tACS did not improve shooting success rates. In this study, shooting success rate was a binary measure of whether the ball passed through the hoop, whereas shooting scores were used to capture subtle performance changes not reflected by shooting success rate alone. Our results, showing no change in shot success rate but an increase in shot score following tACS, indicate that the ball was released closer to the hoop's center, although this did not translate into more successful shots. In other words, the effects of tACS in this study were insufficient for enhancing shooting performance in basketball games, and it is necessary to modify the stimulation frequency and electric field strength to enhance the stimulating effects. The inhibitory interneurons and Purkinje cells in the cerebellar molecular layer are active at 30–80 Hz frequencies (Middleton et al. 2008); thus, frequencies other than 70 Hz should be explored in future studies. Furthermore, the effects of tACS depend on the electric field strength; thus, electrode sites and stimulation intensities that produce higher electric field strengths may enhance the stimulation effects (Kasten et al. 2019). According to Arnold's tongue law, the entrainment effect of tACS increases with higher stimulation intensities, even for oscillatory activities that are further away from the stimulation frequency (Antal and Herrmann 2016). A stimulation intensity of 1.0 mA was used in this study to produce an electric field of 0.0752 V/m. This electric field may have been too weak to improve the shooting success rate. An entrainment effect on a larger population of neurons in the cerebellum may occur using higher stimulation intensities to increase the electric field strength, potentially enhancing the stimulation effects.

This study had several limitations. First, the neurophysiological changes induced by cerebellar tACS are unclear. Second, the stimulation did not improve shooting success rates. Therefore, future studies should investigate neurophysiological changes using techniques such as transcranial magnetic stimulation or electroencephalography to clarify the neural mechanisms of cerebellar tACS and identify more effective stimulation protocols. Third, this study lacks follow‐up data to assess the duration of the stimulation effect. Therefore, it remains unclear whether the observed effects are transient or sustained. Fourth, this study employed nonparametric tests to address data normality, but such tests may have lower statistical power than that of parametric tests and do reveal interaction effects. Finally, all participants were female, and psychological factors (Diotaiuti et al. 2021), such as attention, anxiety, and pressure, were not assessed. Despite these limitations, this study is the first to demonstrate that gamma‐band tACS over the cerebellum improves basketball shooting skills, highlighting new possibilities for tACS to enhance sports performance.

## 5 Conclusion

Our results showed that 70‐Hz tACS over the cerebellum improved basketball shooting skills, indicating that tACS may also enhance sports skills. However, there remains room for improvement in the stimulation effects, and future research should also examine the neural mechanisms involved.

## Author Contributions

**Shota Miyaguchi**: methodology, conceptualization, investigation, formal analysis, writing – original draft, writing – review and editing, funding acquisition. **Yasuto Inukai**: conceptualization; methodology. **Miyu Muroi**: conceptualization, methodology, investigation, formal analysis. **Shunpei Yamamoto**: formal analysis. **Hideaki Onishi**: writing – original draft, writing – review and editing.

## Ethics Statement

The study was approved by the Ethics Committee of the Niigata University of Health and Welfare [approval number 19441‐241213], and the study was conducted in accordance with the principles of the Declaration of Helsinki.

## Consent

Oral and written informed consent was obtained from all participants included in this study.

## Conflicts of Interest

The authors declare no conflicts of interest.

## Peer Review

The peer review history for this article is available at https://publons.com/publon/10.1002/brb3.70943

## Supporting information




**Supporting Materials**: brb370943‐sup‐0001‐SuppMatt.docx

## Data Availability

Datasets generated during and/or analyzed during the current study are available from the corresponding author upon reasonable request.
