# Supplementary material for: Effects of Cerebellar tACS With Gamma Band on Basketball Shooting Skills: A Single‐Blind, Randomized Controlled Trial in College Students With Basketball Experience
Source: Brain Behav. 2025 Oct 15;15(10):e70943. doi: 10.1002/brb3.70943 (PMC12528551; doi:10.1002/brb3.70943)
Supplement: Supplementary file 1 — Supporting Materials: brb370943‐sup‐0001‐SuppMatt.docx [file BRB3-15-e70943-s001.docx]

Phosphenes, Itching, Headache,

Burning sensation, Dizziness

６　 Unbearable

５　Very strong

４　Strong

３　 Moderate

２　Mild

１　 Slight

０　None
